# Evaluation of the Human IgG Antibody Response to *Aedes albopictus* Saliva as a New Specific Biomarker of Exposure to Vector Bites

**DOI:** 10.1371/journal.pntd.0001487

**Published:** 2012-02-21

**Authors:** Souleymane Doucoure, François Mouchet, Sylvie Cornelie, Jean Sébastien DeHecq, Abdul Hamid Rutee, Yelin Roca, Annie Walter, Jean Pierre Hervé, Dorothée Misse, François Favier, Philippe Gasque, Franck Remoue

**Affiliations:** 1 Laboratoire Maladies Infectieuses et Vecteurs: Ecologie, Génétique, Evolution et Contrôle, UMR 224 CNRS/IRD/UM1, Montpellier, France; 2 Direction Régionale des Affaires Sanitaires et Sociale, Saint Denis, La Réunion, France; 3 Centro Nacional de Enfermedades, Santa Cruz, Bolivia; 4 Centre d'Investigation Clinique-Epidémiologie Clinique, Saint Pierre, La Réunion, France; 5 Groupe de Recherche Immunopathologie et Maladie Infectieuses, Saint Denis, La Réunion, France; USAMRIID, United States of America

## Abstract

**Background:**

The spread of *Aedes albopictus*, a vector for re-emergent arbovirus diseases like chikungunya and dengue, points up the need for better control strategies and new tools to evaluate transmission risk. Human antibody (Ab) responses to mosquito salivary proteins could represent a reliable biomarker for evaluating human-vector contact and the efficacy of control programs.

**Methodology/Principal Findings:**

We used ELISA tests to evaluate specific immunoglobulin G (IgG) responses to salivary gland extracts (SGE) in adults exposed to *Aedes albopictus* in Reunion Island. The percentage of immune responders (88%) and levels of anti-SGE IgG Abs were high in exposed individuals. At an individual level, our results indicate heterogeneity of the exposure to *Aedes albopictus* bites. In addition, low-level immune cross-reactivity between *Aedes albopictus* and *Aedes aegypti* SGEs was observed, mainly in the highest responders.

**Conclusion/Significance:**

Ab responses to saliva could be used as an immuno-epidemiological tool for evaluating exposure to *Aedes albopictus* bites. Combined with entomological and epidemiological methods, a “salivary” biomarker of exposure to *Aedes albopictus* could enhance surveillance of its spread and the risk of arbovirus transmission, and could be used as a direct tool for the evaluation of *Aedes albopictus* control strategies.

## Author Summary

*Aedes*-borne viruses like dengue and chikungunya are a major problem in Reunion Island. Assessing exposure to *Aedes* bites is crucial to estimating the risk of pathogen transmission. Currently, the exposure of populations to *Aedes albopictus* bites is mainly evaluated by entomological methods which are indirect and difficult to apply on a large scale. Recent findings suggest that evaluation of human antibody responses against arthropod salivary proteins could be useful in assessing exposure to mosquito bites. The results indicate that 88% of the studied population produce IgG to *Ae. albopictus* saliva antigens in Reunion Island and show that this biomarker can detect different levels of individual exposure. In addition, little cross-reactivity is observed with *Aedes aegypti* saliva, suggesting that this could be a specific marker for exposure to *Aedes albopictus* bites. Taken together, these results suggest that antibody responses to saliva could constitute a powerful immuno-epidemiological tool for evaluating exposure to *Aedes albopictus* and therefore the risk of arbovirus infection.

## Introduction

The incidence of arthropod-borne disease is on the rise and mosquito-borne diseases in particular constitute a world-wide threat [1]. In Asia, Africa and South America, arbovirus diseases are re-emerging, notably dengue and chikungunya. According to the World Health Organization, there are 50 million cases of dengue fever every year and the number of countries declaring cases is increasing [2] Chikungunya is an emerging arbovirus [3] and several outbreaks have been recorded, such as the 2006 epidemic on Reunion Island in the Indian Ocean [4]. The threat of these diseases in the developed world is real with, in addition to the chikungunya outbreak in Italy in 2007 [5], sporadic autochthonous cases of dengue and chikungunya recently reported in Southern France [6]. Therefore, epidemiological tools for evaluating such risks are urgently needed in both developing and developed countries. *Aedes aegypti* and *Aedes albopictus* are both vectors of the dengue and chikungunya viruses, and *Ae. albopictus* populations are dramatically expanding worldwide. Epidemiological evaluation of *Aedes*-borne diseases is currently based on pathogen detection in human populations and entomological methods. The exposure of human populations to *Aedes* is currently evaluated by mapping breeding sites and using mosquito-capture strategies. But these methods have substantial limitations when it comes to large-scale studies in the field, e.g. vector density and transmission risk are estimated by counting immature *Aedes* in breeding sites to derive House and Breteau Indices, a process which is too demanding for regular implementation in the field [7], especially in the urban setting. In addition, current methods for evaluating *Aedes* exposure are mainly applicable at the population level and cannot be used to gauge the heterogeneity of individual exposure. In order to improve vector control and follow the risk of arbovirus transmission, much effort is being devoted to developing new, simple, rapid and sensitive indicators to evaluate human exposure to *Aedes* bites and thus the risk of arbovirus transmission in exposed populations. One promising approach is based on the idea that exposure could be directly assessed by measuring human-vector contact as reflected by the human antibody (Ab) response to arthropod salivary proteins [8]. At the time of biting, the female mosquito injects saliva containing biologically active molecules to favour feeding and some of these are highly immunogenic [9]. Human Ab responses to the saliva of a number of vectors, including *Triatoma* (Chagas disease) [10] and *Phlebotomus* (Leishmaniasis) [11], have been identified as promising biomarkers for vector exposure. Ab responses to the saliva of *Glossina* (the vector of Human African Trypanosomiasis) have been shown to have high diagnostic value [12]. For mosquitoes, Ab responses to whole saliva have been correlated to human exposure to *Culex* mosquitoes [13], and *Anopheles gambiae* [14], *Anopheles dirus* [15] and *Anopheles darlingi* [16], vectors of *Plasmodium*. Recently, it has been shown that the IgG response to whole *An. gambiae* saliva could be a useful biomarker for evaluating the efficacy of malaria vector control [17]. Studies on Ab responses to *Aedes* saliva have tended to focus on human allergic reactions [18] and the identification of the immunogenic proteins [19] although they have shown that quantitative evaluation of anti-saliva Ab responses (IgG and specific isotypes) could give a measure of human exposure to biting *Aedes* [20], [21]. It was recently demonstrated that IgM and IgG responses to *Ae. aegypti* saliva could be used to estimate exposure in transiently exposed populations [22]. Finally, recent data showed that IgE and IgG4 responses to *Ae. aegypti* saliva could be detected in young Senegalese children during the exposure season [23]. The present study addresses one important application of this salivary biomarker as a tool to evaluate the specific exposure of individuals to *Ae. albopictus* bites. Human IgG responses to *Ae. albopictus* saliva (salivary gland extracts; SGE) were measured in adults living on Reunion Island. In this area, *Ae. albopictus* represents the only *Aedes* species which is known to bite humans and is the unique vector of chikungunya. *Ae. aegypti*, which is non anthropophilic in Reunion Island is totally absent from the study area [24]. To check the specificity of this biomarker for *Ae. albopictus*, cross-reactivity was tested by comparing IgG Ab levels i) to *Ae. aegypti* SGE in individuals from Reunion Island and ii) in sera from Bolivian subjects who had only ever been exposed to *Ae. aegypti* species.

## Materials and Methods

### Ethics statement

All studies followed ethical principles as stipulated in the Edinburgh revision of the Helsinki Declaration. The studies in La Reunion and the North of France were approved by a French Ethics Committee (the *Sud Ouest, Outre Mer* Ethics Committee, 25/02/2009) and authorized by the French Drug Agency (AFFSAPS, Ministry of Health; 12/01/2009). The study in Bolivia was approved by the Bolivian Committee of Bioethics (September 2006) and the Institut de Recherche pour le Dévelopement (IRD) “*Comité Consultatif de Déontologie et d'Ethique*” (July 2006). Written informed consent was obtained from every subject.

### Study population

The study populations were from two different areas, namely Reunion Island and Bolivia, for specific exposure to *Ae. albopictus* or *Ae. aegypti*, respectively.

In the south of Reunion Island (Le Tampon), *Ae. albopictus* is abundant and is found up to 1200 meters in winter. Chikungunya transmission was high during the 2006 epidemic [25]. Blood samples were collected in May–June 2009 during the seasonal peak of *Ae. albopictus* exposure, from adults of between 18 and 30 years of age (n = 110). Subjects exposed only to *Ae. aegypti* were randomly selected from a large study conducted in the city of Santa Cruz de la Sierra, Bolivia (n = 104) and pair-matched for age with the Reunion Island subjects. Sera from unexposed individuals (n = 18) in a region free of either *Ae. albopictus* or *Ae. aegypti* (North of France) were used as a negative control.

### Collection of *Aedes* salivary gland extracts

SGE were obtained from 10 day-old uninfected females reared in insectaries. *Ae. albopictus* was bred from larvae collected in the field in Reunion Island (Direction Regionale des Affaires Sanitaires et Sociales, Saint Denis, Reunion Island) and the Bora-Bora strain of *Ae. aegypti* was used (IRD, Montpellier, France). Briefly, two days after a blood meal, the mosquitoes were sedated with CO_2_ and then their salivary glands were dissected out and transferred into a tube containing 30 µl of phosphate buffered saline (PBS). The dissected glands were then pooled in 30 or 60 pairs per batch and frozen at −80°C before extraction. A simple technique consisting of 3 successive freeze-thaw cycles in liquid nitrogen was used to disrupt the membranes. The soluble SGE fraction was then separated by centrifugation for 20 minutes at 30,000 g at +4°C. The concentration of protein was evaluated by the Bradford method (OZ Biosciences) after pooling of the different batches to generate a homogenous SGE for immunological assessment. SGEs were then stored at −80°C before use.

### Evaluation of human IgG Ab levels

An enzyme-linked immunosorbent assay (ELISA) was carried out on Maxisorp plates (Nunc, Roskilde, Denmark) coated with *Ae. albopictus* or *Ae. aegypti* SGE, (0.8 µg/ml PBS) at 37°C for 150 min. Plates were blocked using 250 µl of protein free Blocking-Buffer (Pierce, Thermo Fisher, France) for 60 minutes at room temperature. Individual sera were incubated in duplicate at a 1/100 dilution in PBS-Tween 1%, 4°C overnight. Monoclonal mouse biotinylated Ab against human IgG (BD Pharmingen, San Diego, CA) was incubated at a 1/1000 dilution for 90 minutes at 37°C. Peroxidase-conjugated streptavidin (GE healthcare, Orsay, France) was added at 1/1000 for 60 minutes at 37°C. Colorimetric development was carried out using ABTS (2,2′-azino-bis (3-ethylbenzthiazoline 6-sulfonic acid) diammonium, Pierce) in 50 mM citrate buffer (pH 4) containing 0.003% H_2_O_2_, and absorbance was measured at 405 nm. Each test sample was assessed in duplicate wells and in a blank well containing no antigen (ODn) to measure non-specific reactions, as previously described [17], [23], [26]. Individual results were expressed as the ΔOD value calculated using the equation ΔOD = ODx-ODn, where ODx represents the mean of the OD readings in the two antigen wells. A subject was considered as an “immune responder” if the ΔOD result was higher than the mean ΔOD+(3 SD) for unexposed individuals (negative control). The threshold of positivity was 0.271 for IgG against *Ae. albopictus* and 0.161 for IgG against *Ae. aegypti*.

### Statistical analysis

Graph Pad Prism Software (San Diego, CA USA) was used to analyse the data. After confirmation of non-normal distribution, a non-parametric Mann-Whitney test was used to compare Ab levels between two independent groups, and a non-parametric Kruskal-Wallis test was used for comparisons between more than two groups. A Spearman test was used to assess the correlation between IgG levels against *Ae. albopictus* and *Ae. aegypti* SGEs. All differences were considered significant at p<0.05.

## Results

### IgG response to *Ae. albopictus* to SGE

**Figure 1:**
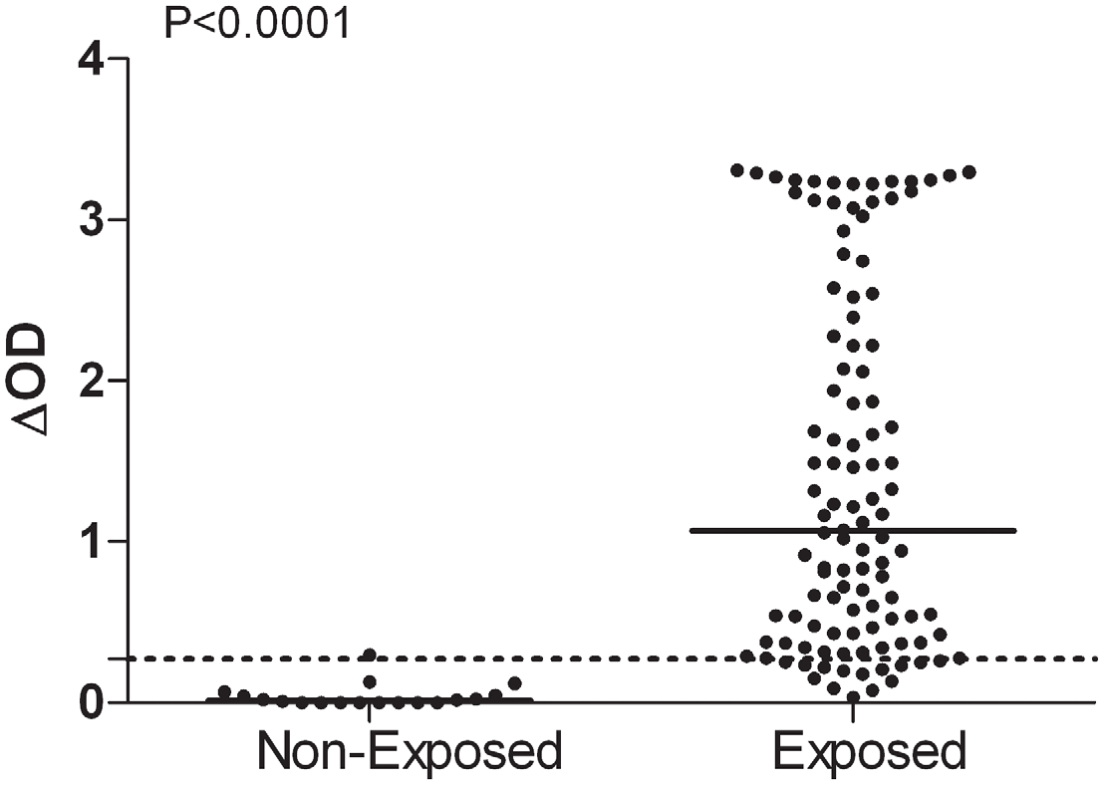
Individual IgG response to *Ae. albopictus* SGE in Reunion Island. Exposed (n = 110, Reunion Island) and unexposed individuals (n = 18, North France). Individual IgG Ab responses are represented by ΔOD. Bars indicate median value in each group and the dotted line represents the threshold of specific Ab response to *Ae. Albopictus* SGE (ΔOD = 0.271).

IgG responses to *Ae. albopictus* SGE were evaluated in individuals from Reunion Island and North of France (Figure 1). In the unexposed group, one individual IgG response was slightly above the cut-off value (ΔOD = 0.297) (Figure 1). In contrast, a high percentage (88%) of the exposed group from La Reunion responded positively to the anti-SGE IgG Ab. Although considerable differences in specific Ab level were observed between exposed individuals (ΔOD from 0.034 to 3.308), a significant difference in specific IgG level was observed between the exposed group (median = 1.067) and the unexposed group (median = 0.015) (p<0.0001, Mann-Whitney test).

### Evaluation of IgG cross-reactivity with *Ae. aegypti* SGE

**Figure 2:**
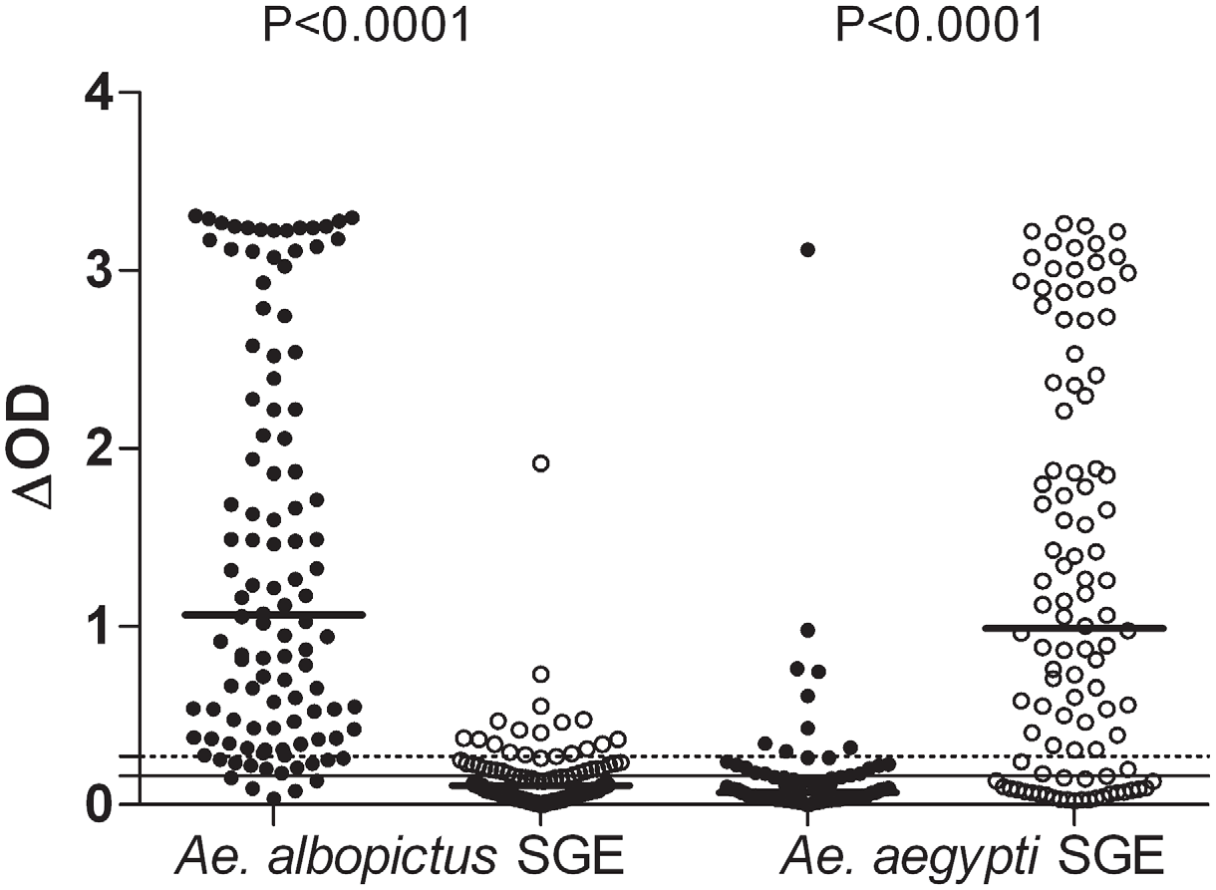
Individual IgG response to *Ae. albopictus* or *Ae. aegypti* SGE in Reunion Island and Bolivia. Individual IgG responses against *Ae. albopictus* SGE and *Ae. aegypti* SGE are presented in individuals from Reunion Island (black circle) and from Bolivia (white circle). The percentage of positive responders is indicated for each group. Bars indicate median value in each group. The dotted and solid lines represent the threshold of specific Ab response to *Ae. albopictus* (ΔOD = 0.271) and *Ae. aegypti* SGE (ΔOD = 0.161), respectively.

Cross-reactivity between *Ae. albopictus* and *Ae. aegypti* SGEs was evaluated by two complementary approaches. First, the specific IgG response to both SGEs (Figure 2) was evaluated in individuals only exposed to *Ae. albopictus* (from Reunion Island); in parallel, IgG responses to both SGEs were assessed in individuals only exposed to *Ae. aegypti* (from Bolivia). In the Bolivian group, 16% showed a positive IgG response against *Ae. albopictus* SGE with only one strong response (ΔOD>1). The median (0.107) level of IgG against *Ae. albopictus* SGE was significantly lower in the Bolivians than in the Reunion Island group (1.067) (P<0.0001, Mann-Whitney test). The IgG response to *Ae. aegypti* SGE was also evaluated in both groups of subjects. As expected, the Bolivian group (exposed only to *Ae. aegypti*) presented high levels of IgG against *Ae. aegypti* SGE with 76% of immune responders. In contrast, only 19% of the subjects from Reunion Island were responders to *Ae. aegypti* SGE and the median (0.068) IgG level was very low compared with the Bolivian group (0.991) (p<0.0001, Mann Whitney test). Only one individual from Reunion presented very high level of IgG to *Ae. aegytpi* SGE (ΔOD>3). In addition, in 58% of double immune responders from Reunion Island, the level of IgG against *Ae. albopictus* SGE was above the 75% percentile value (ΔOD = 2.426; data not shown). In the Bolivian double immune responders, the corresponding figure for *Ae. aegypti* SGE was 50% (ΔOD value of 75% percentile = 2.341). These results indicate that IgG to *Ae. albopictus* and *Ae. aegypti* SGE are cross reactive, particularly in individuals presenting a very high level of IgG.

**Figure 3:**
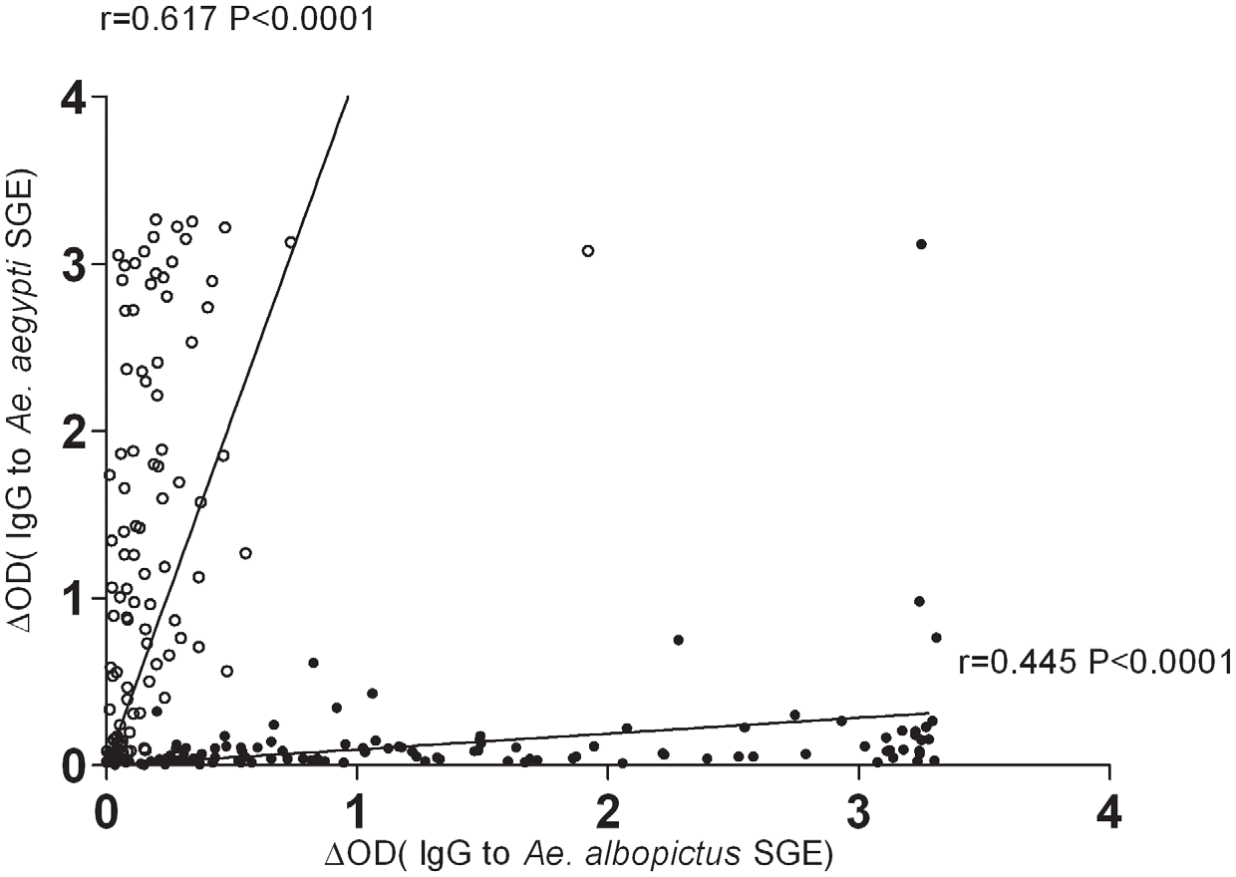
Individual cross-reactivity IgG response between *Ae. albopictus* and *Ae. aegypti* SGE. Correlations between IgG against *Ae. albopictus* SGE and *Ae. aegypti* SGE in Reunion Island (black circle) and Bolivia (white circle). The coefficient of correlation (r) and significance are indicated for each species SGE, black lines represent linear Spearman correlation.

Secondly, the level of specific IgG Ab against both SGEs was compared by a statistical correlation analysis (Figure 3). High positive correlation between IgG against *Ae. albopictus* SGE and *Ae. aegypti* SGE was observed for both the Reunion Island group (r = +0.445; P<0.0001, Spearman test) and the Bolivian group (r = +0.617; P<0.0001, Spearman test). For each population, IgG cross-reactivity was low with few individuals responding to both *Ae. albopictus* and *Ae. aegypti* SGEs. For the Reunion Island group, only 5 individuals showed a strong IgG response to *Ae. aegypti* SGE. These correlations indicate that cross-reactivity between the two *Aedes* species may depend on IgG level. In the Bolivian group, the same trend is observed with only 8 individuals showing a strong IgG response to *Ae. albopictus* SGE.

## Discussion

In this study, we investigated IgG responses to *Ae. albopictus* SGE in exposed adults from Reunion Island where *Ae. albopictus*—the only anthopophilic *Aedes* species—transmits chikungunya. First, specific IgG responses were high in the exposed group and significantly different to those observed in an unexposed population from Europe: 88% of exposed individuals developed IgG against *Ae. albopictus* SGE. In addition, specific IgG Ab levels showed considerable inter-individual variations. Since the intensity of exposure in a given population living in the same area can obviously vary between individuals, these results suggest that the anti-SGE IgG response may be a reliable biomarker for exposure to *Ae. albopictus* bites. Furthermore, the significant difference in Ab levels between unexposed and exposed individuals shows that this biomarker could distinguish individuals exposed to *Ae. albopictus* bites. A useful biomarker for *Ae. albopictus* bites needs to be highly specific and devoid of immune cross-reactivity with other *Aedes* species. We evaluated the cross-reactivity between two species using complementary approaches, i.e. in individuals only ever exposed to *Ae. aegypti* and by analysing IgG levels against both SGEs. In the Bolivian group only exposed to *Ae. aegypti*, 16% of individuals responded to *Ae. albopictus* SGE (heterologous ELISA). The ΔOD values for these “cross-reactive” individuals are characterised by very low IgG levels whereas a high percentage (88%) and high IgG levels (homologous ELISA) were observed in subjects from Reunion Island. In addition, IgG responses against *Ae. aegypti* SGE are significantly different: 76% of immune responders in Bolivia compared with 19% in Reunion Island. Interestingly, we observed that IgG cross-reactivity was mainly detected in high immune responders. In Reunion Island, in 58% of double immune responders, the level of IgG against *Ae. albopictus* SGE was above the third quartile. In parallel, in Bolivia, the level of IgG against *Ae. aegypti* SGE of 50% of double immune responders was above the third quartile. Taken together, these results suggest that there is cross-reactivity between *Ae. albopictus* SGE and *Ae. aegypti* SGE, especially in high immune responders. Further investigations would be required to identify species-specific salivary antigens. IgG response to *Ae. albopictus* SGE has been detected in individuals exposed to the bites of this mosquito [27]. It can be hypothesised that, in Reunion Island, the observed specific IgG responses were elicited as a result of antigenic stimulation following biting by *Ae. albopictus*. In the urban area of Reunion Island, *Ae. albopictus* is highly antropophilic [28] and characterised by numerous “artificial” breeding sites [25] which could explain the high percentage (88%) of specific responders to *Ae. albopictus* SGE. These results point up the relevance of this approach to developing a specific biomarker for exposure to *Ae. albopictus*. However epidemiological factors—history of exposure, genetic background, immune tolerance, etc.—have to be taken into account when explaining variations in responsiveness. Further longitudinal studies could focus on this. To our knowledge, measuring IgGs against *Ae. albopictus* SGE represents the first direct method for evaluating human exposure to *Ae. albopictus* and this parameter probably represents the first genuine biomarker for man-vector contact. This method could help overcome the shortcomings of the current methods which only give indirect measurements of exposure to *Ae. albopictus* and therefore have considerable limitations for evaluating the risk of arbovirus transmission [7]. The current standard methods—immature stage counting and trapping techniques—are both “static” and mainly target “household exposure”, ignoring all the anthropogenic factors that can affect exposure (e.g. water storage practices). *Ae. albopictus* and *Ae. aegypti* are diurnal mosquitoes, biting both indoors and outdoors, and these characteristics may complicate the assessment of exposure using conventional methods. Using anti-SGE IgG responses to evaluate exposure to *Ae. albopictus*, highly heterogeneous Ab levels were observed between exposed individuals, as previously reported for another vector [26]. It could be hypothesized that different levels could reflect the intensity of exposure to biting vectors, e.g. the experimental results indicate that a high Ab response is the result of high exposure and the ame association is also observed for low Ab levels [29]. This has also been observed in human populations in the field where arthropods vectors are endemic. In exposed individuals from endemic areas, it has been shown that the level of anti-saliva Ab was closely associated with the intensity of exposure to vector bites [12], [23], [26], [30]. Therefore, this could be a useful tool for comparing the exposure to *Ae. albopictus* at different sites in a given study area or between different areas, and could be useful for evaluating the efficacy of vector control. In addition, recent findings in the malaria field have shown that Ab responses to saliva antigens are useful in the assessment of low-level exposure to *Anopheles* bites [31]. Detection of low level exposure in newly colonized areas is of particular interest for *Ae. albopictus* due to its ongoing worldwide spread, especially in urban contexts. Moreover, a clear correlation between larval indices and pathogen transmission is difficult to establish when the level of exposure is low [32], [33]. In both cases, evaluation of the anti-saliva IgG response could complement entomological methods. However, several validation steps (e.g. seasonal variation and correlation with entomological measurements) will have to be checked.

In this study, we measured the Ab response to whole SGE. As long as any salivary proteins are shared with other species or genera, the degree of cross-reactivity with major other *Aedes* vectors will have to be assessed. Thus, cross-reactivity was investigated between *Ae. albopictus* and *Ae. aegypti*, closely related species with shared salivary proteins [34], [35]. Only weak cross-reactivity was detected between *Ae. albopictus* and *Ae. aegypti* SGEs, mainly observed in high immune responders. This may suggest that species-specific proteins are more immunogenic than genus-shared proteins. Species-specificity has been already reported with several *Aedes* species [20], [23], [36] and for a broad range of vectors [12], [29], [37]. In contrast, western-blot analysis reveals extensive cross-reactivity and shows that some antigens are common to all *Aedes* species [21], [38], [39]. This low level of cross-reactivity also raises the roles of intensity and history of exposure in determining the acquired IgG response against SGE. Individuals from Reunion Island are unlikely to have been exposed to *Ae. aegypti* because this mosquito is not found in town and its breeding sites are restricted to natural habitats [24]. Cross-reactivity between salivary proteins common to all members of the *Aedes* genus seems therefore to be the most likely explanation of the observed IgG responsiveness to *Ae. aegypti* SGE in Reunion Island. Travelling could also lead to contact with *Ae. aegypti* which is present in most of the islands of the Indian Ocean.

To enhance the usefulness of this biomarker for large-scale applications and to exclude cross-reactivity, an *Ae. albopictus*-specific salivary antigen needs to be identified. In malaria, only one peptide in whole *Anopheles* salivary antigen is an efficient biomarker for exposure to *Anopheles* bites [30]. To improve the sensitivity and the specificity of detection, an immuno-proteomic study is currently underway to identify *Ae. albopictus*-specific proteins and peptides.

The study described here represents the first step for estimating human exposure to *Ae. albopictus* by quantifying the IgG response to vector salivary antigens. In an area of chikungunya transmission, it was shown that the level of Ab against *Ae. albopictus* SGE can be used to identify individuals who have been exposed to the bites of this important vector. Low level cross-reactivity was observed with *Ae. aegypti* SGE suggesting that it will be possible to develop a specific biomarker for human exposure to biting *Ae. albopictus*. By combining the use of such a biomarker with classical entomological and epidemiological methods, it could enhance the assessment of human exposure to *Ae. albopictus* and therefore contribute to both accurate prediction of the risk of arbovirus transmission and evaluation of the efficacy of vector control.
